# Recurrent Adult Sacrococcygeal Teratoma Developing Adenocarcinoma: A Case Report and Review of Literatures

**DOI:** 10.1155/2021/5045250

**Published:** 2021-11-27

**Authors:** Shengjie Cui, Jing Han, Binny Khandakar, Barak Friedman, Domingo Nunez, Gabriel A. Sara, Gabriel S. Levi

**Affiliations:** ^1^The Department of Pathology, Molecular and Cell-Based Medicine, Icahn School of Medicine at Mount Sinai, 1 Gustave L. Levy Pl, New York, NY 10029, USA; ^2^Department of Diagnostic, Molecular and Interventional Radiology, Icahn School of Medicine at Mount Sinai, 1 Gustave L. Levy Pl, New York, NY10029, USA; ^3^The Department of Surgery, Icahn School of Medicine at Mount Sinai, 1 Gustave L. Levy Pl, New York, NY 10029, USA; ^4^The Department of Medicine, Hematology and Medical Oncology, Icahn School of Medicine at Mount Sinai, 1 Gustave L. Levy Pl, New York, NY 10029, USA

## Abstract

Sacrococcygeal teratomas (SCT) are most commonly seen in infants and children but are rare in adults. Most adult SCT are benign and mature with a minority of tumors having immature components or overt malignancy. Here, we report a 65-year-old female with a SCT developing adenocarcinoma. The patient was diagnosed with benign sacrococcygeal cystic teratoma on her initial hospital visit and was treated with surgical resection. She was followed up postoperatively and was noted to have a markedly elevated CA 19-9 level 13 months after the surgery. Radiological and clinical examination revealed thickening of the perirectal soft tissues, located near the inferior portion of her previous incision site. Histological evaluation of the lesion showed invasive, moderately differentiated adenocarcinoma. Immunohistochemical staining results were suggestive, but not diagnostic, of anal gland adenocarcinoma. This case report expands the knowledge regarding an adenocarcinoma arising from a previously benign, adult SCT.

## 1. Introduction

Teratomas are germ cell tumors, composed of cells derived from one or more germ cell layers. Teratomas of the sacrococcygeal region are most commonly seen in neonates with an approximate prevalence of 1/27,000 live births [1], predominantly affecting females [1, 2]. However, they are extremely rare in adults. Most of the sacrococcygeal teratomas (SCT) are benign and mature; malignant transformation within adult SCT is exceedingly rare with only several case reports documented [3–9]. Most of the adult SCT are located within the pelvis and may cause compressive symptoms such as lower back pain, bowel/urinary dysfunctions, and venous engorgement of the lower limbs [10]. The diagnosis depends on radiological imaging and histopathologic analysis. Early and complete surgical resection relieves the patient's symptoms and normally leads to a favorable prognosis. However, there is an approximately 15% recurrent rate; risk factors for recurrence include incomplete resection, immature component, and frank malignancy [11, 12].

## 2. Case Description

**Figure 1:**
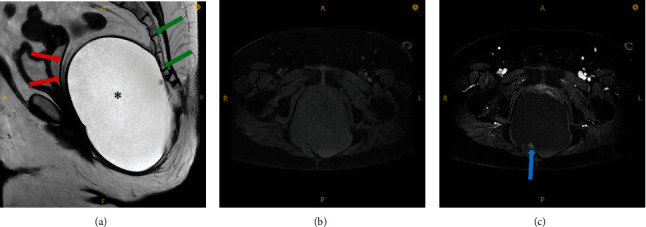
Pelvic MRI of the tumor showing a large retrorectal cystic mass with solid mural nodule. A sagittal T2 weighted pelvic MRI (a) demonstrated a large predominantly cystic mass (∗) which is anterior to the sacrum and coccyx (green arrow) and posterior to the rectum (red arrow). An unenhanced fat suppressed T1 weighted axial image (b) and a gadolinium enhanced fat suppressed T1 weighted axial image (c) demonstrated an enhancing nodule (blue arrow) in the posterior portion of the mass.

**Figure 2:**
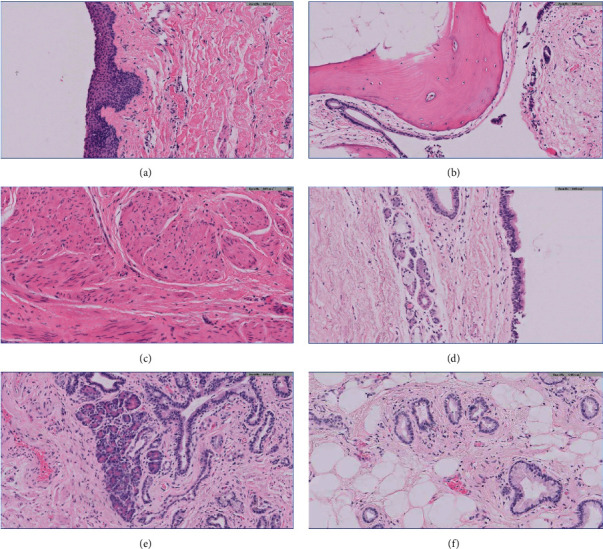
Microscopic examination of the cyst wall revealing squamous epithelium (a), mature bone (b), smooth muscle (c), respiratory epithelium (d), pancreatic tissue (e), and enteric epithelium (f).

**Figure 3:**
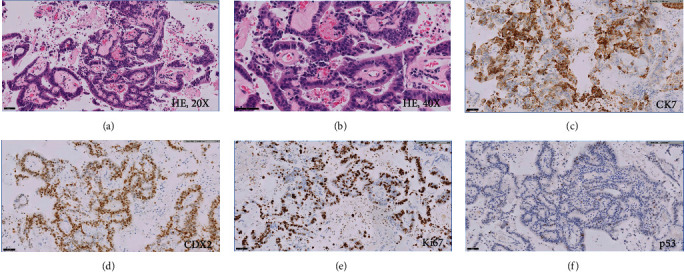
Proliferating glandular structures with interspersed vasculature (a); cellular atypia was appreciated at 40X magnification (b). Immunostains of the atypical glandular epithelium were positive for CK7 (c) and CDX2 (d). The Ki67 proliferative index was high (e). The p53 was wild-type (f).

**Figure 4:**
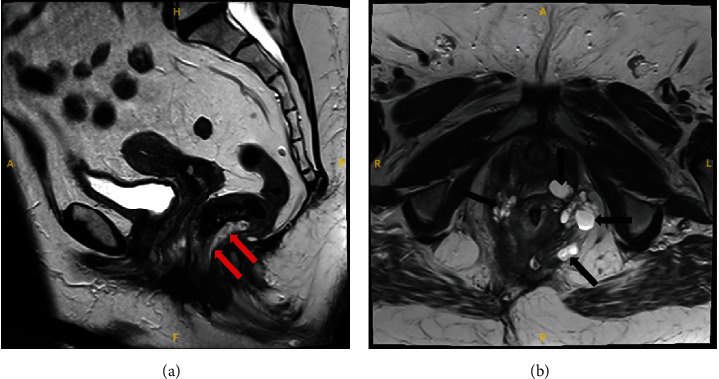
Pelvic MRI 13 months later with a sagittal T2 weighted image (a) and an axial T2 weighted image (b) demonstrated a T2 hyperintense mass within the posterior wall of the low rectum (red arrow) and multiple additional T2 hyperintense perirectal masses (black arrow).

**Figure 5:**
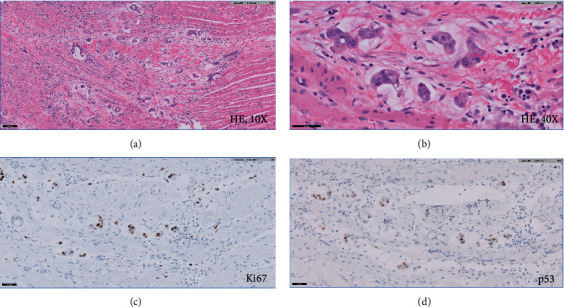
Microscopic examination of the recurrent tumor showing invasive, moderately differentiated adenocarcinoma. The tumor cell islands displayed an infiltrative pattern at 10x magnification (a), with marked structural and cellular atypia demonstrated at 40x magnification (b). The malignant glands showed a high proliferative index on Ki-67 (c), while p53 was wild-type (d).

The patient is a 65-year-old female who initially presented to our hospital in 2019, complaining of pelvic pressure and anorectal pain for 2 years. She also had difficulty with urination and altered bowel movements. Abdominal and pelvic computed tomography (CT) scan showed a 17 cm cystic retrorectal mass. Pelvic magnetic resonance imaging (MRI) revealed a 17.3 × 11.4 × 7.7 cm retrorectal cystic mass with solid mural nodule (Figure 1). Her preoperative CEA level was 3.7 ng/mL (normal range, <5 ng/mL), and the CA-19-9 was elevated at 266 U/mL (normal range, <37 U/mL). The patient received surgical resection via Kraske approach with resection of the involved coccyx. At surgery, she was found to have a large cystic neoplasm encompassing the entire space above the levators and extending superiorly to the pelvic floor and posteriorly to the coccyx. The tumor was densely adherent to the surrounding tissues as well as the wall of the rectum and contained brownish fluid. At pathologic examination, the cyst wall was rough and uneven with a wall thickness ranging from 0.2 to 0.8 cm. Microscopic examination of the tumor showed squamous epithelium (ectoderm), mature bone, smooth muscle (mesoderm), respiratory epithelium, pancreatic tissue, and enteric epithelium (endoderm) (Figures 2(a)–2(f)). Noticeably, the tumor also demonstrated foci of proliferating glandular structures with interspersed vasculature (Figure 3(a)); cellular atypia was appreciated at high magnification (Figure 3(b)). Immunostains showed the atypical glandular epithelium to be positive for CK7 and CDX2 (Figures 3(c) and 3(d)) and focally positive for CK20, suggestive of enteric/colonic differentiation. Despite a high Ki67 index (Figure 3(e)), the histology of the tumor lacked definite evidence of malignancy and the p53 was wild-type (Figure 3(f)). Overall, the above evaluations confirm the diagnosis of benign mature SCT. The patient was followed up postoperatively and was noted to have a markedly elevated CA 19-9 level (397.8 U/mL) 13 months after the prior surgery. She also had rectal and low back pain which prompted surveillance with a CT scan of the abdomen and pelvis showing thickening of the perirectal soft tissues. This was confirmed on a follow-up MRI of the abdomen and pelvis (Figure 4). On physical exam, she was noted to have a subcentimeter palpable mobile, hard, rubbery nodule in right inner subcutaneous fat tissues of the left buttock. PET/CT (not shown) demonstrated intensely FDG avid nodularity in the perianal region as well as along the peritoneal reflection involving the pelvic floor, pelvic sidewalls extending superiorly up to the presacral region which favors local recurrence. She was subsequently brought to the operating room and the perirectal soft tissue mass, which was near the inferior portion of her previous Kraske incision in the right buttock, was removed. The lesion was sent for frozen section, which consisted of yellow tan fibro-fatty tissue measuring 2.3 × 1.5 × 1.5 cm; the diagnosis was that of recurrent sacrococcygeal teratoma cannot exclude underlying malignancy. Permanent sections of the lesion revealed invasive, moderately differentiated adenocarcinoma (Figure 5), arising from the preexisting sacrococcygeal teratoma. The tumor cell islands displayed an infiltrative pattern at low magnification (Figure 5(a)), with marked structural and cellular atypia appreciated at high magnification (Figure 5(b)). In comparison to the patient's previous excision (Figures 3(a) and 3(b)), these findings showed a similar histology but with a greater degree of nuclear atypia and definite invasive architecture. The malignant glands showed a high proliferative index on Ki-67 (Figure 5(c)), while p53 was still wild-type (Figure 5(d)). Additional immunostains of the invasive glands were reactive for CK7, AE1/AE3, Ber-EP4, and CK19, while nonreactive for CK20, CDX2, GCDFP-15, TTF-1, PAX8, PAX2, GATA3, WT-1, and vimentin. This immunophenotype was nonspecific but suggestive of anal gland adenocarcinoma. She was scheduled for chemotherapy with FOLFOX (Leucovorin, Oxaliplatin followed by 5FU) given every 14 days × 12 cycles.

## 3. Discussion

Teratomas are composed of cells derived from one or more germ cell layers (ectoderm, mesoderm, and endoderm), and they are classified as a subgroup of germ cell tumors. Different theories exist regarding the origin of the SCT, including that they are derived from the totipotential cells in Hensen's node of the primitive knot [13]. The majority of teratomas are located in the ovaries or testicles (gonadal teratomas); however, they can also develop in midline structures such as mediastinum, retroperitoneal space, presacral and sacrococcygeal areas, and brain/spine [14–16].

SCT are classified according to the Altman classification system [17]/American Academy of Pediatrics Surgical Section:

1. Type I: the tumor is predominantly external with a very minimal intrapelvic component.
2. Type II: the tumor is predominantly external but has a significant intrapelvic extension.
3. Type III: the tumor is visible externally, but is predominantly located in the pelvic area with extension into the abdomen.
4. Type IV: the tumor has no external presentation and is located in the pelvic bone.

In neonates and children, type I tumors have the lowest risk of malignancy and type IV tumors have the highest risk of malignancy [18].

Most neonatal SCT are externally visible, while most adult SCT present as a pelvic mass and often cause compressive symptoms such as lower back pain, bowel/urinary dysfunctions, and venous engorgement of the lower limbs [10]. Sometimes, SCT can present as an anal fissure when associated with infection [19]. Interestingly, this patient was found to have anal fissure in 2018 during perianal exam, which could be the early presentation of her SCT. Macroscopically, SCT can be cystic, solid, or a mixture of solid and cystic components. Histopathologically, SCT are classified into three categories: mature, immature, and malignant. Mature teratomas are benign and contain fully differentiated somatic tissues such as the epithelium, muscles, and bones. Immature teratomas consist of at least foci of embryonal structures or incompletely differentiated tissue components; primitive neuroectodermal structures are often present [20]. Teratomas containing any malignant elements are considered to be malignant. In adults, there are only a few case reports noting malignant transformation from sacrococcygeal/presacral teratomas [3–9]: two cases being mucinous adenocarcinoma, one case being adenocarcinoma of gastrointestinal origin, and four cases being nonspecific adenocarcinoma. Cytogenetic aberrations (amplifications of 8q and 12p) have been reported to be associated with malignant transformation to an adenocarcinoma [4]. Elevated serum tumor markers (CEA, CA19-9, AFP, and HCG) are suggestive of malignant transformation and could be useful to monitor postoperative recurrence [6, 10, 21, 22].

The differential diagnosis of SCT in adults includes tailgut cyst, chordoma, meningocele, pilonidal cysts, rectal duplication or anal gland cysts, osteomyelitis of sacrum, giant cell tumor of sacrum, perirectal abscess, fistula, granuloma, and tuberculosis [7, 23]. Our patient had a cystic lesion with histology showing components from all three germ cell layers, leading to a diagnosis of benign mature sacrococcygeal cystic teratoma. Noticeably, foci of atypical glandular structures were also identified in the teratoma, with a high Ki67 proliferative index. She was monitored after initial surgery and at 13 months, her CA 19-9 level was markedly elevated. Radiological imaging favored local recurrence, and excisional biopsy was performed. Histology revealed invasive, moderately differentiated adenocarcinoma. Immunostains in the invasive glands were reactive for CK7, AE1/AE3, and Ber-EP4 and nonreactive for CK20, CDX-2, and GCDFP15. This immunophenotype was suggestive but not diagnostic of anal gland adenocarcinoma [24]. The atypical glandular structures observed in the previously resected tumor did not meet the criteria of adenocarcinoma; however, these atypical features may act as a a histological indication of the tumor's ability to recur and even transform into adenocarcinoma.

Early and complete surgical excision of SCT with coccygectomy is the mainstay of treatment. The coccyx may contain the nidus of totipotential cells; the risk of tumor recurrence is reported to be 30-40% without concomitant excision of the coccyx [7]. Unlike gonadal teratomas, SCT are often unencapsulated, making it difficult to achieve a complete resection. The overall recurrence rate for mature teratomas is about 10%, and that for immature teratomas is about 20% [12, 25]. Complications of surgery include massive bleeding, bowel/urinary dysfunction, and dysesthesia [26]. For malignant cases, additional treatment with chemotherapy and/or radiotherapy is required. Given the rarity of the entity, the standard treatment regimen has not been well established. Benign SCT have excellent prognosis with complete surgical excision; however, the prognosis of malignant SCT is poor [27].

## 4. Conclusion

Adult SCT are rare and often recognized following symptoms caused by compressive symptoms, including lower back pain, bowel/urinary dysfunctions, and venous engorgement of the lower limbs. Most cases are mature and benign; however, rarely malignant transformation can occur. Atypical structures with a high Ki67 proliferative index, if present, may help to alert that tumor recurrence or transformation into adenocarcinoma is possible. The diagnosis relies on radiological imaging (CT or MRI), serum markers, and histopathologic analysis. Complete surgical excision with coccygectomy is the mainstay treatment for benign cases (both mature and immature SCT) and often leads to a favorable prognosis. Malignant SCT require surgical excision with adjuvant therapy including chemotherapy and/or radiotherapy. Postoperative follow-up of the patients is crucial to detect recurrence.
